# Endoscopic full-thickness plication for the treatment of gastroesophageal reflux after peroral endoscopic myotomy: a randomized sham-controlled study

**DOI:** 10.1055/a-2040-4042

**Published:** 2023-03-21

**Authors:** Amit Maydeo, Gaurav Patil, Nagesh Kamat, Ankit Dalal, Amol Vadgaonkar, Sanil Parekh, Rajen Daftary, Sehajad Vora

**Affiliations:** 1 Baldota Institute of Digestive Sciences, Gleneagles Global Hospital, Mumbai, Maharashtra, India

## Abstract

**Background** Endoscopic full-thickness plication (EFTP) has shown promising results in gastroesophageal reflux disease (GERD), but its efficacy in GERD after peroral endoscopic myotomy (POEM) is unclear.

**Methods** In a prospective, randomized trial of post-POEM patients dependent on proton pump inhibitors (PPIs) for documented GERD, patients underwent EFTP (plication to remodel the gastroesophageal flap valve) or an endoscopic sham procedure (positioning of the EFTP device, but no stapling). The primary end point was improvement in acid exposure time (AET) < 6 % (3 months). Secondary end points included improvement in esophagitis (3 months), GERD Questionnaire (GERDQ) score (3 and 6 months), and PPI usage (6 months).

**Results** 60 patients were randomized (30 in each group). At 3 months, a significantly higher proportion of patients achieved improvement in AET < 6 % in the EFTP group compared with the sham group (69.0 % [95 %CI 52.1–85.8] vs. 10.3 % [95 %CI 0–21.4], respectively). EFTP was statistically superior to sham (within-group analysis) in improving esophageal AET, DeMeester Score, and all reflux episodes ( *P* < 0.001). A nonsignificant improvement in esophagitis was noted in the EFTP group ( *P* = 0.14). Median GERDQ scores (3 months) were significantly better ( *P* < 0.001) in the EFTP group, and the same trend continued at 6 months. A higher proportion of patients in the sham group continued to use PPIs (72.4 % [95 %CI 56.1–88.7] vs. 27.6 % [95 %CI 11.3–43.8]). There were no major adverse events in either group.

**Conclusion** EFTP improved post-POEM GERD symptoms, 24-hour pH impedance findings with normalization in one-third, and reduced PPI usage at 6 months.

## Graphical abstract

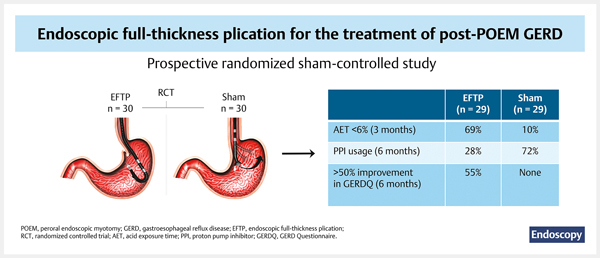

## Introduction

Peroral endoscopic myotomy (POEM) is one of the established treatments for achalasia 1 2 , but post-POEM gastroesophageal reflux disease (GERD) remains a concern 3 4 . Post-POEM patients have a low incidence of symptomatic GERD, despite endoscopic evidence of esophagitis and abnormal acid exposure time (AET) 3 5 6 . However, asymptomatic GERD can predispose to complications such as strictures, Barrett’s metaplasia/dysplasia, and even esophageal cancer 7 . Medical therapy for GERD is limited by ongoing long-term costs, dependence, and potential side effects 8 . Endoluminal therapies were introduced to enhance antireflux mechanisms at the lower esophageal sphincter 9 ; however, they have failed to show consistent symptomatic improvement or durable treatment response.

The literature on post-POEM reflux is limited but growing, with multicenter studies mentioning AET in about 50 % of patients, and symptomatic GERD and erosive esophagitis in about 10 % of patients 10 11 . In the current era, regardless of variation in the reported incidence of post-POEM GERD, it is undeniable that reflux after POEM affects a sizeable number of patients, and the clinical implications are yet to be determined, with potential long-term complications. Unlike laparoscopic Heller myotomy 12 , POEM does not include an associated antireflux procedure. Recently, transoral incisionless fundoplication 13 and endoscopic full-thickness plication (EFTP) have been gaining increasing popularity for the treatment of GERD 14 15 16 . However, their efficacy in post-POEM reflux is unclear. We conducted a randomized, sham-controlled study of EFTP in post-POEM patients with GERD.

## Methods

### Study population

Consecutive patients with achalasia who underwent POEM over 5 years, from May 2013 to April 2018, were assessed for study eligibility. We performed a prospective randomized participant- and assessor-blind, sham-controlled study to evaluate the effectiveness of EFTP using a new EFTP device (GERDx; G-SURG GmbH, Seeon-Seebruck, Germany) in post-POEM patients from June 2019 to August 2021. The first and last patients were enrolled in June 2019 and February 2021, respectively, and the follow-up of the last patient was in August 2021. The study protocol was reviewed and approved by the Institutional Ethics Committee (IEC/OA-39/19). Written informed consent was obtained from each patient before enrollment. The co-investigators who collected the data (G.P., A.V.) and analyzed the data (P.J.) were unaware of the study group assignments. The trial was registered in a publicly accessible database before recruitment of the first patient.

### Eligibility

Inclusion criteria were patients aged > 18 years who had undergone POEM (by posterior myotomy, Eckardt score < 3), and had ≥ 6 months of proton pump inhibitor (PPI)-dependent GERD (GERD questionnaire [GERDQ] score ≥ 8), abnormal 24-hour pH impedance off PPIs (AET > 6 %), and willingness to cooperate with postoperative follow-up assessment. Patients were excluded if any of the following applied: previously failed POEM, sigmoid esophagus, hiatus hernia > 2 cm, grade D erosive esophagitis, esophageal ulcerations and strictures, Barrett’s esophagus, cirrhosis of the liver, chronic kidney disease, pregnancy or plans for pregnancy in the next 6 months, plans to travel during the study period, receipt of psychotropic drugs (anxiolytics, antidepressants), coagulation disorders, prior gastric or esophageal surgery, body mass index < 18.5 kg/m ^2^ , American Society of Anesthesiologists (ASA) physical status > II, and refusal to provide consent.

### Symptom evaluation

Patient demographics were entered into a predesigned form. At initial screening, symptom assessment was performed using a validated questionnaire, GERDQ 17 , and the requirement for anti-secretory medicines (detailed drug history) was assessed along with upper gastrointestinal endoscopy. All anti-secretory medications were stopped 7 days prior to assessment. At upper gastrointestinal endoscopy, esophagitis (Los Angeles classification scale) 18 and Hill grading of the gastroesophageal junction (GEJ) flap valve were assessed 19 .

Esophageal high resolution manometry (Trace 1.2.3a V software; Royal Melbourne Hospital, Melbourne, Australia) and a 24-hour pH impedance study were performed in all patients at baseline. The latter was done with the ZepHr Impedance/pH Reflux Monitoring System (Sandhill Scientific, Highlands Ranch, Colorado, USA), and was visually checked for inaccuracy at 3-minute intervals by an expert reader who had experience of reading more than 1000 multichannel intraluminal impedance pH studies. The recording time was at least 24 hours. The total number of reflux episodes, DeMeester score, and total AET were noted. The results were considered abnormal if the esophageal AET was ≥ 6 % and was calculated for each patient after excluding abnormal readings 20 21 . Esophageal acid normalization was defined as AET < 4 %. The maximum timeframe between 24-hour pH impedance results and EFTP or sham procedure while considering the patient for study inclusion was 2 weeks.

### Assignment

Patients were randomly assigned by permuted block randomization (six blocks) to undergo either the EFTP interventional procedure or an endoscopic sham procedure, with a target allocation ratio of 1:1. Computer-generated randomization assignments were obtained before study enrollment by a statistician who was not part of the study. Individual patient assignments were prepared (N.K.) in sequentially numbered opaque sealed envelopes. A numbered envelope was drawn (A.M.) at the time of the procedure from a set of sealed envelopes containing the allocation. Patients were unaware of their group assignment. Patient blinding was lifted once 6 months’ follow-up was completed.

### Preprocedural requirements

All patients had to undergo routine laboratory investigations for anesthesia assessment. Patients were asked to continue their previous medications as appropriate, doses of which were unaltered.

### Interventions

Procedures were performed under general anesthesia with the patients in a supine position. A smaller-sized endotracheal tube was used and placed on the left corner of the mouth. Antibiotic prophylaxis (amoxycillin 1000 mg + clavulanic acid 200 mg) was given. Premedications included ondansetron (4 mg) and PPI (pantoprazole 40 mg).

### Endoscopic full-thickness plication

**Fig. 1:**
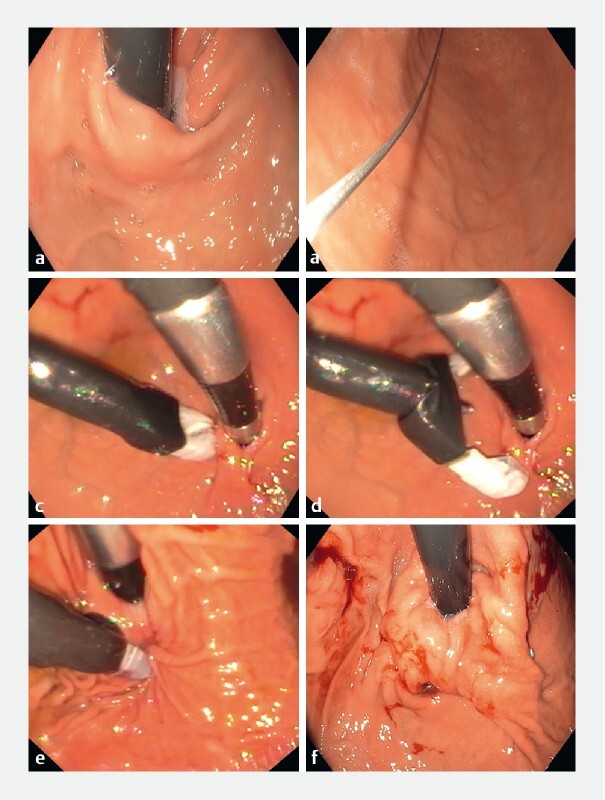
Endoscopic full-thickness plication (EFTP) procedure. **a** Lax lower esophageal sphincter after peroral endoscopic myotomy. **b** A guidewire was placed into the stomach. **c** The EFTP device was retroflexed at the stomach cardia. **d** Both arms of the EFTP device were opened and the helix was introduced. **e** Full-thickness plication was performed at the cardia. **f** Two full-thickness plications were performed to achieve remodeling of the gastroesophageal junction.

EFTP was performed using the GERDX system by a single endoscopist (A.M.) in patients allocated to the endoscopic plication group. After careful examination of the esophagus and stomach, a guidewire with a long flexible tip (G-SURG GmbH) was placed into the stomach antrum using a gastroscope (GIF-HQ190; Olympus, Tokyo, Japan). The device was prepared by attaching the disposable staple on one arm and the receiving pledget on the opposite instrument arm. The arms of the device were then closed, and then the loaded instrument (distal end lubricated) was introduced over the guidewire into the stomach. Air was then insufflated into the stomach using the attached inflating cuff. An ultrathin gastroscope (GIF-XP190N; Olympus) was then passed through the channel of the EFTP device and retroflexed to view the GEJ (S.V.). The device arms were then opened and positioned using rotatory as well as to and fro movements. A screw-tipped tissue retractor (helix) was then pushed forward through the center of the open arms and to at least 1 cm below the Z line in the gastric mucosa along the greater curvature. Once in the correct position, the tissue helix was rotated clockwise to enter the stomach wall. The tissue retractor was then withdrawn to pull the tissue between the open arms of the EFTP device. The device arms were then closed to fire the staples through the pulled stomach wall ( Fig. 1 ). After this, the tissue retractor was first unrotated, and the tissue was released. The device arms were then opened, and the device was disconnected from the stapled tissue by a rotatory and pushing movement. The device and the ultrathin endoscope were removed after unlocking all the wheels. This step was repeated, and a second stapling was performed as described above. The second implant was placed about 0.5 cm away from the first implant. The device was again removed, and a normal gastroscope was reintroduced to evaluate the plication at the remodeled GEJ.

### Sham technique

The sham procedure (done in the same endoscopy suite) was identical to the treatment procedure by positioning the retroflexed device below the GEJ; however, staples were not applied. The scope was maneuvered with multiple repositioning and rotations for 20–25 minutes (A.M., S.V.) to simulate an EFTP procedure.

### Post-intervention monitoring

Immediately after the intervention, patients were placed on a soft diet and analgesics as appropriate. PPI (pantoprazole 40 mg) was given parentally on the day of the procedure and then oral pantoprazole 40 mg/day was prescribed with instruction to be taken 30 minutes before breakfast for the next week, then discontinued. Patients were discharged 24 hours after the procedure and were asked to maintain a medication diary. The requirement and need for medication were enquired about over the telephone and recorded (G.P.). Patients with reflux symptoms for two consecutive days were allowed to take antacids on demand. For inadequate symptom control, PPI (pantoprazole 40 mg) was added after telephone consultation and documented. All patients were treated similarly before and after the procedure.

### Follow-up

All patients received two telephone reminders for the scheduled follow-up (± 3 days). The scheduled follow-up visit was carried out at 1, 3, and 6 months unless the patient had any new symptoms. At 3 months, 24-hour pH impedance and upper gastrointestinal endoscopy were performed in all patients (see **Table 1 s** in the online-only Supplementary material). During this visit, patients were assessed clinically, and the GERDQ was completed. At 6 months, patients were interviewed for symptoms and a GERDQ was completed. The interview ( **Appendix 1 s** ) was done by a senior consultant (G.P.). The total duration of involvement by each patient (either group) in the study was 6 months after randomization.

### End points

The primary end point was treatment success, defined as an improvement in AET to < 6 % on 24-hour pH impedance from baseline to 3 months in the intention-to-treat population. Secondary end points included improvement in the GERDQ score by > 50 %, requirement for PPI, and healing of erosive esophagitis. In the event of symptom recurrence, patients were asked to contact the study site. Treatment failure was defined as the need for resumption of PPI therapy (for at least three consecutive weeks) as assessed at clinic visits and/or if patients had post‐procedure complaints (dysphagia or chest pain) that required the EFTP procedure to be revised within 2 weeks after the procedure. Patients from the sham group classified as ‘treatment failure’ were offered EFTP after completing the 6-month follow-up and followed thereafter according to clinical practice.

### Safety

The occurrence and consequences of adverse events were recorded. Adverse events were considered mild (sore throat, heartburn, mild epigastric pain, shoulder pain, vomiting, or bloating), moderate (dysphagia, chest pain, severe reflux symptoms), or severe (events requiring hospitalization, emergency surgery, bleeding requiring blood transfusion, sepsis, organ failure, or death) 22 .

### Sample size calculation

The sample size was estimated for the comparison of two proportions 23 24 25 26 . Assuming equal sample size allocation, 5 % level of significance, and 80 % power at an estimated proportion of improvement at 3 months post-intervention in 24-hour pH impedance findings (AET < 6 %) of 70 % in EFTP and 32 % in the sham group, the sample size was 26 per group. After correcting for a 15 % dropout rate at follow-up in each group, the minimum sample size required to observe a statistically significant difference was 30 per group.

### Minor changes to methods after trial commencement

For study inclusion, patients had to be negative for SARS-CoV-2 with a reverse-transcriptase polymerase chain reaction respiratory tract sample and radiologically (computed tomography) to exclude Covid-19 pneumonia. The study duration was initially planned for 15 months but was extended by 12 months. No changes were made to the study protocol.

### Statistical analysis

The patient details were anonymized and analyzed with IBM Statistical Package for the Social Sciences (SPSS) for Windows version 24.0, Professional (IBM Corp., Armonk, New York, USA). The normality of the data was analyzed (P.J.) through the Shapiro–Wilk test. Descriptive statistics are used for continuous variables, and frequency and percentage are reported for categorical variables. Comparison between GERDQ scores was made using Friedman Repeated Measures Analysis of Variance by Ranks. The chi-squared test was used for nonparametric nominal data. The nonparametric tests, Mann–Whitney *U* test (between group) and Wilcoxon signed-rank test (within group), were used to compare the distribution in the two groups. Data, if skewed, were subjected to logarithmic transformation. The estimates were back converted, and interpretation was made in terms of geometric mean. Repeated measures analysis of variance was used to determine the longitudinal changes in GERDQ scores across the time points between the groups. Results of 24-hour pH impedance and GERDQ are graphically represented in box plots. GERDQ values are presented with an error bar graph at the 95 %CI and mean difference. Observed data of PPI use in the two groups are shown as 3-D stacked bar graphs. A *P* value of < 0.05 was considered statistically significant.

## Results

**Fig. 2:**
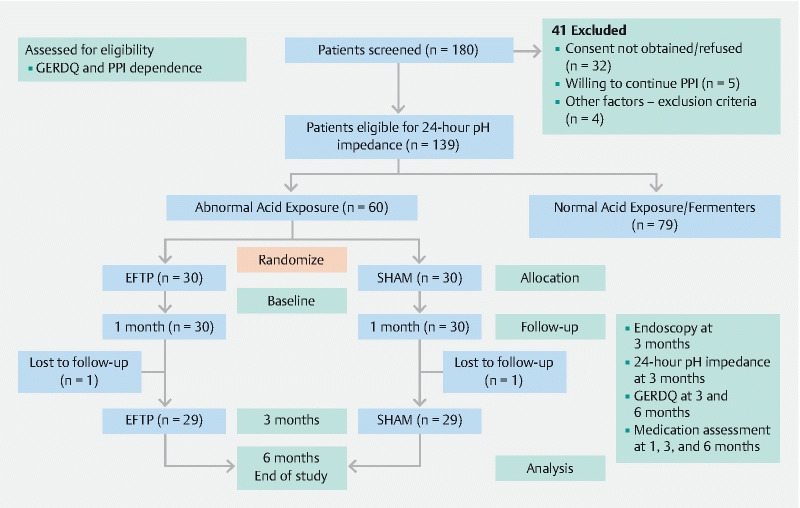
CONSORT flow diagram 27 . EFTP, endoscopic full-thickness plication; GERDQ, Gastroesophageal Reflux Disease Questionnaire; PPI, proton pump inhibitor.

Of the 139 screened patients who were eligible for inclusion, 60 were randomized (30 in each group) and received the intended treatment (EFTP/sham) and were analyzed for the primary end point without deviations from the randomization protocol ( Fig. 2 ). Of the randomized patients, 58 (96.7 %) completed the 3- and 6-month follow-up assessments. Two patients were excluded from the final analysis due to loss to follow-up and missing patient information. The baseline characteristics in the two groups were comparable ( Table 1 ).

**Table 1:** Baseline characteristics of the two groups.

Variable	EFTP (n = 30)	Sham (n = 30)	*P*
Age, median (IQR), years	40.5 (33.7–45)	40 (35.7–45.2)	0.63
Male sex, n (%)	17 (56.7)	16 (53.3)	0.80
BMI, median (IQR), kg/m ^2^	26.5 (24.9–28.2)	25.7(24.6–26.6)	0.11
Post-POEM GERD history, n (%)			0.64
6–12 months	21 (70.0)	18 (60.0)	
> 12 months	9 (30.0)	12 (40.0)	
Median (IQR), months	16.5 (12.0–21.2)	17.5 (14.0–21.2)	
GERDQ score, median (IQR)	11 (9–12)	9 (8–12)	0.23
Percent time pH < 4.0, median (IQR)	28.6 (24.6–35.6)	27.5 (24.6–32.1)	0.56
DeMeester scores, median (IQR)	53.9 (37.1–64.5)	52.3 (36.7–62.9)	0.71
Total reflux episodes, median (IQR)	148.0 (120.2–178.2)	140.5 (122.0–177.2)	0.64
Acid reflux episodes, median (IQR)	59.0 (55.7–70.5)	62.5 (50.5–68.2)	0.90
Nonacid reflux episodes, median (IQR)	89.5 (62.7–109.7)	82 (60.5–109.5)	0.92
Endoscopy (esophagitis LA grade), n (%)			0.74
Normal	2 (6.7)	3 (10.0)	
A	15 (50.0)	18 (60.0)	
B	5 (16.7)	4 (13.3)	
C	8 (26.7)	5 (16.7)	
Hill’s grade of gastroesophageal flap valve, n (%)			0.60
I	12 (40)	14 (46.7)	
II	18 (60)	16 (53.3)	
Daily PPI use, n (%)	30 (100)	30 (100)	> 0.99

EFTP, endoscopic full-thickness plication; IQR, interquartile range; POEM, peroral endoscopic myotomy; GERD, gastroesophageal reflux disease; GERDQ, GERD Questionnaire; LA, Los Angeles; PPI, proton pump inhibitor.

### Ambulatory pH study

**Fig. 3:**
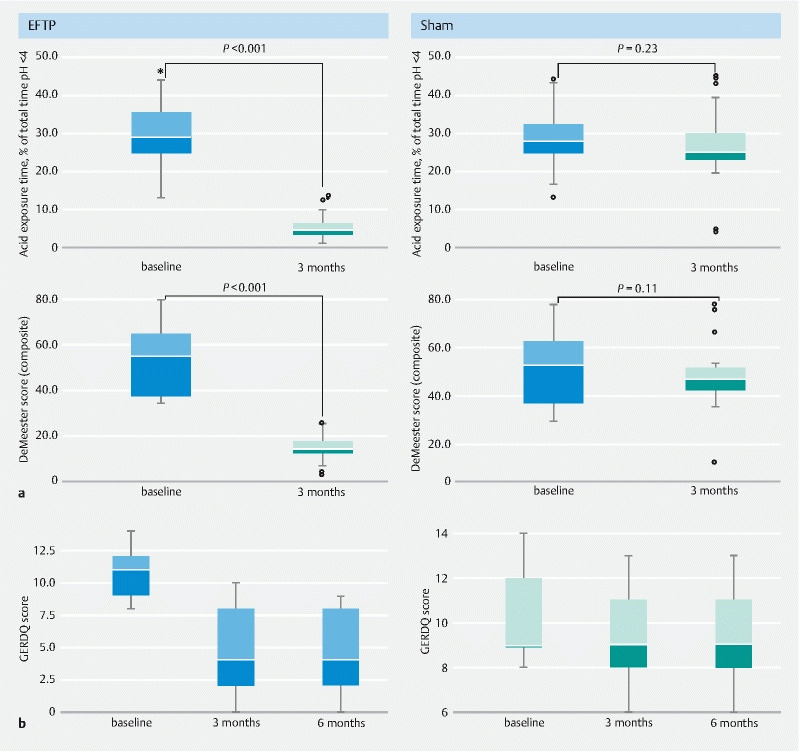
Outcomes of endoscopic full-thickness plication compared with sham. **a** 24-hour pH impedance findings. **b** Gastroesophageal Reflux Disease Questionnaire (GERDQ).

At 3 months, a significantly higher proportion of patients in the EFTP group compared with the sham group achieved the primary end point of AET < 6 % (69.0 % [95 %CI 52.1–85.8] vs. 10.3 % [95 %CI 0–21.4], respectively) ( Table 2 ). The majority of patients in the EFTP group achieved improvement (> 50 % from baseline) in AET (96.6 % [95 %CI 89.9–100]) compared with few patients in the sham group (13.8 % [95 %CI 1.2–26.3]). Only 11 patients (37.9 %) in the EFTP group compared with none in the sham group achieved esophageal acid normalization ( Table 2 ). The median total number of reflux episodes and median DeMeester score significantly improved in the EFTP group compared with the sham group ( *P* < 0.001) ( Table 3 , Fig. 3a ). The EFTP group was statistically superior (within-group analysis) to the sham group in improving distal esophageal AET, DeMeester Score (composite), and all reflux episodes (total) (acid reflux and nonacid reflux) ( *P* < 0.001).

**Table 2:** Comparison of primary and secondary end points in the two groups.

Variable	EFTP n = 29	Sham n = 29
AET < 6 %, 3 months (Primary endpoint)		
Achieved, n (%)	20 (69.0)	3 (10.3)
95 %CI	(52.1–85.8)	(0–21.4)
50 % reduction in AET at 3 months		
Achieved, n (%)	28 (96.6)	4 (13.8)
95 %CI	(89.9–100)	(1.2–26.3)
Normalization of AET < 4 %, 3 months		
Achieved, n (%)	11 (37.9)	0 (0)
95 %CI	(20.3–55.6)	(0–0)
Daily PPI usage 3 months		
Yes, n (%)	9 (31.0)	19 (65.5)
95 %CI	(14.2–47.9)	(48.2–82.8)
Daily PPI usage 6 months		
Yes, n (%)	8 (27.6)	21 (72.4)
95 %CI	(11.3–43.8)	(56.1–88.7)
GERDQ improvement by > 50 %, 6 months		
Achieved, n (%)	16 (55.2)	0 (0)
95 %CI	(37.1–73.3)	(0–0)

EFTP, endoscopic full-thickness plication; AET, acid exposure time, PPI, proton pump inhibitor, GERDQ Gastroesophageal Reflux Disease Questionnaire.

**Table 3:** Comparison of esophageal 24-hour pH impedance findings at baseline and 3 months after the procedure.

Variable	EFTP	Sham	Between-group comparison
Percent time pH < 4.0			
Baseline, median (IQR)	28.6 (24.6–35.6)	27.5 (24.6–32.1)	
3 months, median (IQR)	4.7 (3.4–6.5)	24.9 (21.7–30.2)	< 0.001
Within-group comparison	< 0.001	0.23	
DeMeester Score			
Baseline, median (IQR)	53.9 (37.1–64.5)	52.3 (36.7–62.9)	
3 months, median (IQR)	14.1 (11.6–18.7)	46.9 (41.6–52.1)	< 0.001
Within-group comparison	< 0.001	0.11	
Reflux episode activity			
Baseline, median (IQR)	148.0 (120.2–178.2)	140.5 (122.0–177.2)	
3 months, median (IQR)	45 (24–80)	134 (106–156)	< 0.001
Within-group comparison	< 0.001	0.08	
Acid reflux episodes			
Baseline, median (IQR)	59.0 (55.7–70.5)	62.5 (50.5–68.2)	
3 Months, median (IQR)	16.0 (10.5–35.0)	54.0 (45.0–62.5)	< 0.001
Within-group comparison	< 0.001	0.07	
Nonacid reflux episodes			
Baseline, median (IQR)	89.5 (62.7–109.7)	82.0 (60.5–109.5)	
3 months, median (IQR)	31.0 (14.5–46.5)	80.0 (53.5–91.0)	< 0.001
Within-group comparison	< 0.001	0.30	

Reflux episodes detected by impedance categorized as acid or nonacid by pH.

### Symptom assessment by GERDQ

The median GERDQ scores showed a similar improvement and were significantly better (6 months) in the EFTP group compared with the sham group (4 [IQR 2–8] vs. 9 [IQR 8–11], respectively; *P* < 0.001) ( Fig. 3b ). At 6 months, GERDQ improvement by > 50 % was seen in 16 patients (55.2 %) vs. none in sham group ( Table 2 ). At 6 months, 21/29 patients (72.4 %) had a GERDQ score of < 8 in the EFTP group compared with 5 (17.2 %) in the sham group. GERDQ scores were subjected to logarithmic transformation. There was a significant change in the GERD scores from baseline through 6 months ( *P* < 0.001), as well a significant difference noted between the groups ( *P* < 0.001) ( **Table 2 s** , **Table 3 s** , **Fig. 1 s** , **Fig. 2 s** ).

### Esophagogastroduodenoscopy

All patients in the EFTP group underwent successful endoscopic plication with the adequate remodeling of the GEJ, with each patient receiving two staples. The EFTP procedure was performed in a median of 25 minutes (range 24–35), with the remodeling of the gastroesophageal flap valve of adequate length and circumference, resulting in a direct reduction of the Hill grading of the hiatus hernia. Hills grade 1 was achieved in 26 patients (89.6 %) after the EFTP procedure ( **Table 4 s** ).

At 3 months in the EFTP group, endoscopy showed a maintained omega-shaped GEJ flap valve in 26/29 patients (89.6 %) with good adherence to the scope. Although esophagitis grades were better in the EFTP group, the improvement in esophagitis was not statistically significant ( *P* = 0.14). (POEM provides symptomatic relief from achalasia but has no effect on esophageal peristalsis).

### Medication use

At 3 months, a significantly higher proportion of patients in the sham group than in the EFTP group were using PPI (65.5 % [95 %CI 48.2–82.8] vs. 31.0 % [95 %CI 14.2–47.9], respectively) ( Table 2 , **Fig. 3 s** ). At 6-month follow-up, a significantly higher proportion of patients in the sham group than in the EFTP group continued to use PPI (72.4 % [95 %CI 56.1–88.7] vs. 27.6 % [95 %CI 11.3–43.8], respectively) ( Table 2 , **Table 4 s** , **Fig. 3 s** ).

### Adverse events

Four patients (13.8 %) in the EFTP group had mild adverse events ( **Table 5 s** ). Nonspecific chest pain and left shoulder pain (mild) were treated conservatively with analgesics. In one patient who complained of pain and dysphagia (Eckardt score 4) in the first week of the procedure, endoscopy showed the nonabsorbable staple passing across the GEJ within the esophageal lumen with edema around the GEJ. The staple was cut using a loop cutter (Olympus) and no further interventions were done. In the sham group, two patients (6.9 %) had mild adverse events: one (3.4 %) had nausea (mild) and the other (3.4 %) had a sore throat (mild). No patients in either group had any other serious adverse events. There was no recurrence of achalasia symptoms after EFTP.

## Discussion

In this study, at 3 months, patients randomized to EFTP had achieved the primary end point with a significant reduction in esophageal AET < 6 %. They also showed improvement in GERDQ score and reduction in PPI usage up to the 6-month follow-up, with no major adverse events. These prospective data suggest that symptom improvement and healing of esophagitis are not attributed to a sham effect. A partial, rather than complete, fundoplication is achieved during EFTP, which compares favorably with total fundoplication for controlling GERD but causes less postoperative dysphagia. The stapling enhances the angle of His and preserves the mucosal flap valve mechanism.

Endoluminal therapies for GERD have been utilized for more than two decades. EFTP creates an effective antireflux barrier by introducing an implant/suture material into the area of the lower esophageal sphincter, thereby altering the compliance of the sphincter 15 . A single plicator implant in initial studies was replaced by multiple plicator implants for better outcomes. Following the encouraging results of previous studies, Weitzendorfer et al. 16 showed an improvement in quality of life, reflux symptoms, and DeMeester scores in 30 patients (75 %) at 3 months with the new EFTP device. Our results align with earlier reports that showed EFTP to be effective in GERD ( **Table 6 s** ). EFTP may act as a rescue between PPI and laparoscopic fundoplication. The benefits of POEM over laparoscopic Heller myotomy with a partial fundoplication may be outweighed by the high incidence of GERD. Therefore, post-POEM patients with symptoms requiring PPIs are ideal candidates for minimally invasive endoscopic interventions. If post-POEM GERD can be controlled by adding an endoscopic antireflux procedure in selected patients, then it would tip the balance strongly toward POEM as the procedure of choice for patients with achalasia. The decision to perform an antireflux procedure is governed by various factors. Any tissue structure remodeling would have occurred by about 3–4 months post-POEM. The symptom duration and severity of GERD symptoms despite an adequate dose of PPI for about 6 months post-POEM seem appropriate criteria for an antireflux procedure.

The published studies with plicator showed that the majority of adverse events were mild and resolved spontaneously . Hoarseness, cough, shoulder pain, and abdominal pain were reported. The latter is caused by trauma to the gastric cardia and the esophagus due to the placement of a second or third plicator implant. If the staple is placed too close to the distal esophagus, it can cause luminal narrowing and patients can present with dysphagia. In patients who have loose cardia diameter, 2–3 plications placed along a slightly diagonal vector recreates the normal antireflux valve by re-establishing the angle of His. The suture material and its length can impair the gastric/esophageal tissue. Tissue structure, deeper tissue penetration of the suture, and the way these implants are deployed can lead to bleeding and hematoma formation. As only 30 patients in this study received the EFTP procedure, conclusions regarding adverse events and safety of EFTP should be considered as exploratory, and long-term data are still required. Unlike surgical fundoplication, extra gastric mobilization of the fundus to release the tension of the fundoplication cannot be achieved with EFTP, and the continuous force on the repair eventually causes the anatomy of the GEJ to return to its original shape, causing the flap valve to unravel. This could be why eight patients (27.6 %) in the EFTP group continued to consume PPI.

The rate of post-POEM GERD depends on the type of measurement, and there is a significant difference between symptoms, endoscopic evidence, and pH measurement 36 37 . GERD post-POEM is frequently asymptomatic, but leads to more severe esophagitis. Regardless of pH-positive GERD post-POEM, the symptoms are milder and reflux symptom association is poor despite more severe esophagitis 38 . Furthermore, approximately 60 % of patients with abnormal AET or evidence of esophagitis have no symptoms 39 40 . Measuring clinical symptomatology and severity of GERD can be a complex issue in patients with achalasia because of impaired peristalsis, food stasis, and fermentation. This could possibly explain the difference between GERD symptoms, PPI consumption, and esophagitis rate in the two study arms.

The study has some strengths. This was the first randomized sham-controlled trial that demonstrated an improvement in GERD symptoms, improvement in AET, and a decreased requirement for PPI usage among post-POEM patients in whom the majority of the antireflux endoluminal therapies have shown only modest effects. This improvement in AET looks clinically relevant.

The study also has some limitations. First, the study is from a single center, the results of which may not be generalizable. Although achalasia cardia can occur at any age, patients usually receive a diagnosis between the ages of 25 and 60 years but more commonly between 40 and 60 years. The patients in this study had a median age of about 40 years, a median body mass index of 26 kg/m ^2^ , and were mainly ASA I and II. Second, a single experienced endoscopist performed all the procedures. As the investigator was not blinded, an element of performance bias would have perhaps influenced the results. The site had previously successfully performed EFTP in 16 patients; hence there was no learning curve. Third, the follow-up period was only up to 6 months. Laparoscopic fundoplication for de novo GERD patients or concomitant laparoscopic fundoplication with Heller myotomy has shown diminishing durability over time 41 . Therefore, the ideal study period for assessment of procedure durability should perhaps have been a minimum of 1 year. Fourth, quality of life was not assessed. The short-term outcomes of EFTP with the new device should therefore serve as a reference for future studies evaluating long-term results.

In conclusion, this prospective, randomized sham-controlled trial showed that EFTP using a new plication device was safe and effective in managing post-POEM GERD. Endoscopic therapy led to a significant reduction in PPI use, and improvement in GERD symptoms and 24-hour pH impedance findings. The validity of the study findings needs greater consideration in future multicenter randomized controlled studies with a larger patient cohort, and if the results are reproducible, EFTP could become a suitable endoscopic modality for treatment of post-POEM GERD.

## Tables 1 s–6 s, Figs. 1 s–3 s, Appendix 1 s

Supplementary material Zusatzmaterial
