# Supplementary material for: Endoscopic full-thickness plication for the treatment of gastroesophageal reflux after peroral endoscopic myotomy: a randomized sham-controlled study
Source: Endoscopy. 2023 Mar 21;55(8):689–98. doi: 10.1055/a-2040-4042 (PMC10374353; doi:10.1055/a-2040-4042)
Supplement: Supplementary file 1 — Supplementary material [file 22408supmat_10-1055_a-2040-4042.pdf]

Supplementary material

**Endoscopic full-thickness plication for the treatment of gastroesophageal reflux after peroral endoscopic myotomy: a randomized sham-controlled study**

Amit Maydeo, Gaurav Patil, Nagesh Kamat, Ankit Dalal, Amol Vadgaonkar, Sanil Parekh, Rajen Daftary, Sehajad Vora

**Table 1s** - Study design

| Details | Pre-procedure | Post-procedure (EFTP and sham) | | |
|---|---|---|---|---|
| | Baseline | 1 month | 3 months | 6 months |
| | Off PPI | | | |
| Clinical history | √ | √ | √ | √ |
| Esophageal manometry | √ | - | - | - |
| Esophagogastroduodenoscopy | √ | - | √ | - |
| GERD-Q questionnaire | √ | - | √ | √ |
| GERD medication assessment | - | √ | √ | √ |
| Esophageal 24 h pH impedance | √ | - | √ | - |
| Telephonic reminder for scheduled follow up | - | √ | √ | √ |

**Table 2s - Comparison of GERDQ scores at baseline, 3 and 6 months after the procedure**

| **Variable** | **EFTP** | **Sham** | **Between group comparison** |
|---|---|---|---|
| GERDQ Median (IQR) | | | |
| Baseline (n=60) | 11 (9, 12) | 9 (8, 12) | |
| 3 Months (n=58) | 4 (2, 8.5) | 9 (8, 11) | <0.001 |
| 6 Months (n=58) | 4 (2, 8) | 9 (8,11) | <0.001 |
| Within group comparison | <0.001 | 0.66 | |

GERDQ, Gastroesophageal reflux disease questionnaire. Medians report first and third quartiles
IQR, interquartile range

 © 2023. Thieme. All rights reserved.

Supplementary material

**Table 3s - Logarithmic transformation of GERDQ variables and use of repeated measures ANOVA to assess between-group differences**

| Variable | **Group** | **Mean** | **SD** |
|---|---|---|---|
| lnGERDQ baseline | EFTP | 2.35 | 0.17 |
| | Sham | 2.30 | 0.19 |
| | Total | 2.33 | 0.18 |
| lnGERDQ 3 months | EFTP | 1.47 | 0.72 |
| | Sham | 2.22 | 0.19 |
| | Total | 1.85 | 0.64 |
| lnGERDQ 6 months | EFTP | 1.44 | 0.67 |
| | Sham | 2.22 | 0.20 |
| | Total | 1.84 | 0.62 |

| Within Subjects Effect | Mauchly's W | Approx. Chi-Square | df | Sig. | Epsilon[b] | | |
|---|---|---|---|---|---|---|---|
| | | | | | Greenhouse-Geisser | Huynh-Feldt | Lower-bound |
| factor1 | .159 | 99.136 | 2 | <0.001 | 0.543 | 0.556 | 0.500 |

| Source | | Type III Sum of Squares | df | Mean Square | F | Sig. |
|---|---|---|---|---|---|---|
| factor1 | Sphericity Assumed | 9.027 | 2 | 4.513 | 59.216 | .000 |
| | Greenhouse-Geisser | 9.027 | 1.087 | 8.307 | 59.216 | <0.001 |
| | Huynh-Feldt | 9.027 | 1.112 | 8.119 | 59.216 | .000 |
| | Lower-bound | 9.027 | 1.000 | 9.027 | 59.216 | .000 |
| factor1 * Group | Sphericity Assumed | 6.227 | 2 | 3.113 | 40.850 | .000 |
| | Greenhouse-Geisser | 6.227 | 1.087 | 5.730 | 40.850 | <0.001 |
| | Huynh-Feldt | 6.227 | 1.112 | 5.601 | 40.850 | .000 |
| | Lower-bound | 6.227 | 1.000 | 6.227 | 40.850 | .000 |

Within subject effect: P<0.001, Interaction effect: P<0.001

| Source | Type III Sum of Squares | df | Mean Square | F | Sig. |
|---|---|---|---|---|---|
| Intercept | 687.610 | 1 | 687.610 | 1702.550 | .000 |
| Group | 10.448 | 1 | 10.448 | 25.870 | <0.001 |
| Error | 22.213 | 55 | .404 | | |

Between subject effect: P<0.001

As GERDQ scores were not normally distributed, they were subjected to logarithmic transformation and the estimates after the analysis were back converted, and interpretation was made in terms of geometric mean. Analysis revealed a significant change in the scores from baseline through six months (p<.001). Further, there was a significant difference between the groups (p<.001). More importantly, the interaction term was also significant (p<.001), which means that the change across the time points was not the same in both the groups (Fig. 2 s profile plot).

© 2023. Thieme. All rights reserved.

## Supplementary material

**Table 4s - Comparison of endoscopic parameters and PPI usage in the two groups (off PPI)**

| Variable | EFTP (n=29) 3 months | Sham (n=29) 3 months | P |
|---|---|---|---|
| Primary endpoint AET <6% | 20(69%) | 3(10.3%) | <.001 |
| 50% reduction from baseline in AET | 28(96.6%) | 4(13.8%) | <.001 |
| AET <4% | 11(37.9%) | 0 | <.001 |
| Endoscopy (esophagitis LA grade), n (%) | | | |
| Normal | 12(41.3%) | 5(17.2%) | 0.137 |
| A | 14(48.3%) | 18(62.1%) | |
| B | 3(10.3%) | 4(13.8%) | |
| C | 0 | 2(6.9%) | |
| Hill’s grade of gastroesophageal flap valve, n (%) | | | |
| I | 26(89.6%) | 16(55.2%) | 0.003 |
| II | 3(10.4%) | 13(45.8%) | |
| Daily PPI use, n (%) | 9(31.03%) | 19(65.5%) | 0.009 |
| | EFTP (n=29) | Sham (n=29) | |
| | 6 months | | |
| Daily PPI use, n (%) | 8(27.6%) | 21(72.4%) | 0.001 |
| GERDQ >50% improvement | 16(55.2%) | 0 | <.001 |

AET acid exposure time, PPI proton pump inhibitor, GERDQ Gastroesophageal reflux disease questionnaire

 | © 2023. Thieme. All rights reserved.

## Supplementary material

**Table 5s - Adverse events following the procedure**

| No | Adverse events EFTP | Age/Gender | Post POEM GERD history (months) | Days of Hospitalization | Treatment Details | PPI | Follow up |
|---|---|---|---|---|---|---|---|
| 1 | Nausea, Shoulder Pain | 41/ Male | 10 | 3 | Treated with analgesics and PPI | Off PPI | Completed 6 months |
| 2 | Nausea | 28/ Female | 10 | 2 | No intervention, PPI was continued | Off PPI | Completed 6 months |
| 3 | Dysphagia, Shoulder Pain, Abdominal pain | 35/ Male | 15 | 4 | Underwent Balloon dilation, Treatment failure | No details available | Lost to follow up after 2 months |
| 4 | Chest Pain, Shoulder Pain | 46/ Male | 16 | 4 | Treated with analgesics | On PPI | Completed 6 months |
| Adverse events SHAM | | | | | | | |
| 1 | Throat Pain, Sore throat | 40/ Male | 25 | 2 | Antacids (Gaviscon) | No details available | Lost to follow up after 1 month |
| 2 | Nausea | 36/ Male | 8 | 2 | No intervention, PPI was continued | Off PPI | Completed 6 months |

 | © 2023. Thieme. All rights reserved.

Supplementary material

**Table 6s - Outcome of studies with the old and new full-thickness plicator devices for GERD**

| Study, Year | No of Centers | Country | Study Design | No of Patients | Device | Follow up | QOL used | Patients off PPI | Adverse events |
|---|---|---|---|---|---|---|---|---|---|
| Chuttani R. et al, 2003 [1] | Single | INDIA | Pilot Study | 6 | Plicator (NDO Surgical, Inc., Mansfield, MA) | 12 months | GERD-HRQL | 50% | Mild mid epigastric pain (33.3%), difficulty in eructation's (16.7%) |
| Rothstein R. et al, 2006 [2] | Multicenter (Seventeen) | USA EUROPE | RCT | 78 | Plicator (NDO Surgical, Inc., Mansfield, MA) | 3 months | GERD-HRQL | 50% (ITT) | 39(50%) had one or more events. Pharyngolaryngeal pain (9%), Retrosternal/chest pain or discomfort (12%), Epigastric pain or discomfort (12%), Radiating shoulder pain (12%), Abdominal pain or discomfort (9%), Nausea (8%), Hospitalizations (6%), Vomiting (5%), Dysphagia / Leukocytosis / Pneumoperitoneum / Eructation (3% each) |
| Pleskow D. et al, 2008 [3] | Multicenter (Seven) | USA | Prospective | 33 | Plicator (NDO Surgical, Inc., Mansfield, MA) | 5 years | GERD-HRQL | 33% | Sore throat (45%), abdominal pain (41%) and chest pain (24%). Transient dysphagia (21%), dyspnea (6.1%), |

 | © 2023. Thieme. All rights reserved.

# Supplementary material

| | | | | | | | | | |
|---|---|---|---|---|---|---|---|---|---|
| | | | | | | | | | mucosal abrasion in the fundus (3%) |
| Jeansonne LO. et al, 2009 [4] | Single | GEORGIA | Prospective | 58 | Plicator (NDO Surgical, Inc., Mansfield, MA) | 8.1 months (mean) | - | None | - |
| Renteln VD. et al, 2009 [5] | Multicenter (Four) | GERMANY | Prospective | 41 | Plicator (Ethicon Endosurgery, Sommerville, NJ, USA) | 12 months | GERD-HRQL | 69% | Abdominal pain (44%), shoulder pain (24%), and chest pain (17%) |
| Koch O. et al, 2013 [6] | Single | AUSTRIA | Prospective | 36 | Plicator (Ethicon Endosurgery, Sommerville, NJ, USA) | 12 months | Gastrointestinal Quality of Life Index | 50% | Post procedure bleeding (2.7%) |
| Weitzendorfer M. et al, 2018 [7] | Single | GERMANY | Prospective | 40 | New GERDx device, (G-SURG GmbH, Seeon-Seebruck, Germany) | 3 months | Gastrointestinal Quality of Life Index | 63.3% | Sore throat (20%), chest pain (17.5%), hematoma at the GE junction, pneumonia with pleural effusion, Suture passing left hepatic lobe and pleural empyema, Mallory–Weiss–lesion at GE junction |

 | © 2023. Thieme. All rights reserved.

## Supplementary material

| | | | | | | | | | |
|---|---|---|---|---|---|---|---|---|---|
| Kaindlstorfer A et al, 2013 [8] | Single | GERMANY | RCT | 37 | Plicator (Ethicon Endosurgery, NDO Surgical, Inc., Mansfield, MA) | 3 months | Gastrointestinal Quality of Life Index | 64.5% | Postprocedural gastric bleeding from the plication site -required transfusion, endoscopic reinforcement of the plication by additional pledgeted sutures |
| Antoniou SA et al, 2012 [9] | Single | AUSTRIA | Randomized study | 29 | Plicator (NDO Surgical, Mansfield, MA) | 12 months | Gastrointestinal Quality of Life Questionnaire | 48% | - |
| Domagk D et al, 2006 [10] | Single | GERMANY | RCT | 26 | Plicator (Bard EndoCinch, Wilson-Cook Endoscopic Suturing Device and NDO Plicator) | 6 months | Gastrointestinal Quality of Life Index | 77% | Reinforcement of the plication |

 | © 2023. Thieme. All rights reserved.

Supplementary material

References

1. Chuttani R, Sud R, Sachdev G et al. A novel endoscopic full-thickness plicator for the treatment of GERD: A pilot study. Gastrointest Endosc 2003;58:770-776
2. Rothstein R, Filipi C, Caca K et al. Endoscopic full-thickness plication for the treatment of gastroesophageal reflux disease: A randomized, sham-controlled trial. Gastroenterology 2006;131:704-712
3. Pleskow D, Rothstein R, Kozarek R et al. Endoscopic full-thickness plication for the treatment of GERD: Five-year long-term multicenter results. Surg Endosc 2008;22:326-332
4. Jeansonne LO 4th, White BC, Nguyen V et al. Endoluminal full-thickness plication and radiofrequency treatments for GERD: an outcomes comparison. Arch Surg 2009;144:19-24
5. von Renteln D, Schiefke I, Fuchs KH et al. Endoscopic full-thickness plication for the treatment of gastroesophageal reflux disease using multiple Plicator implants: 12-month multicenter study results. Surg Endosc 2009;23:1866-1875
6. Koch OO, Kaindlstorfer A, Antoniou SA et al. Subjective and objective data on esophageal manometry and impedance pH monitoring 1 year after endoscopic full-thickness plication for the treatment of GERD by using multiple plication implants. Gastrointest Endosc 2013;77:7-14
7. Weitzendorfer M, Spaun GO, Antoniou SA et al. Clinical feasibility of a new full-thickness endoscopic plication device (GERDx) for patients with GERD: results of a prospective trial. Surg Endosc 2018;32:2541-2549
8. Kaindlstorfer A, Koch OO, Antoniou SA et al. A randomized trial on endoscopic full-thickness gastroplication versus laparoscopic antireflux surgery in GERD patients without hiatal hernias. Surg Laparosc Endosc Percutan Tech 2013;23:212-222
9. Antoniou SA, Koch OO, Kaindlstorfer A et al. Endoscopic full-thickness plication versus laparoscopic fundoplication: a prospective study on quality of life and symptom control. Surg Endosc 2012;26:1063-1068
10. Domagk D, Menzel J, Seidel M et al. Endoluminal gastroplasty (EndoCinch) versus endoscopic polymer implantation (Enteryx) for treatment of gastroesophageal reflux disease: 6-Month results of a prospective, randomized trial. Am J Gastroenterol 2006;101:422–430

 | © 2023. Thieme. All rights reserved.

## Supplementary material

**Fig. 1s** Mean plot along with error bars for GERDQ score between EFTP and SHAM

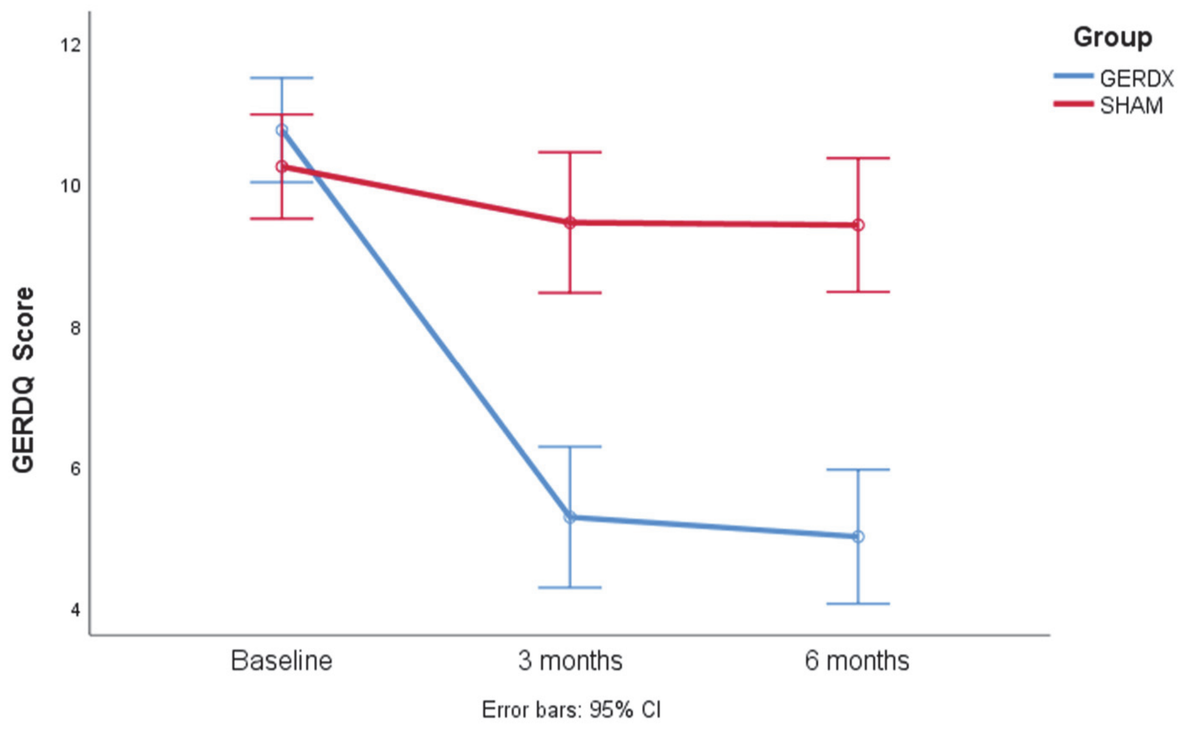


**Fig. 2s** Profile plot comparing the estimated marginal means of a dependent variable (GERDQ)

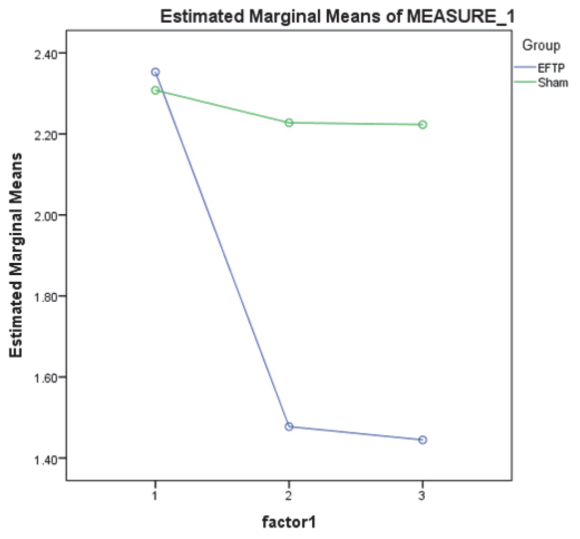


 | © 2023. Thieme. All rights reserved.

## Supplementary material

**Fig. 3s** Proton pump inhibitor use at baseline, 3 and 6 months

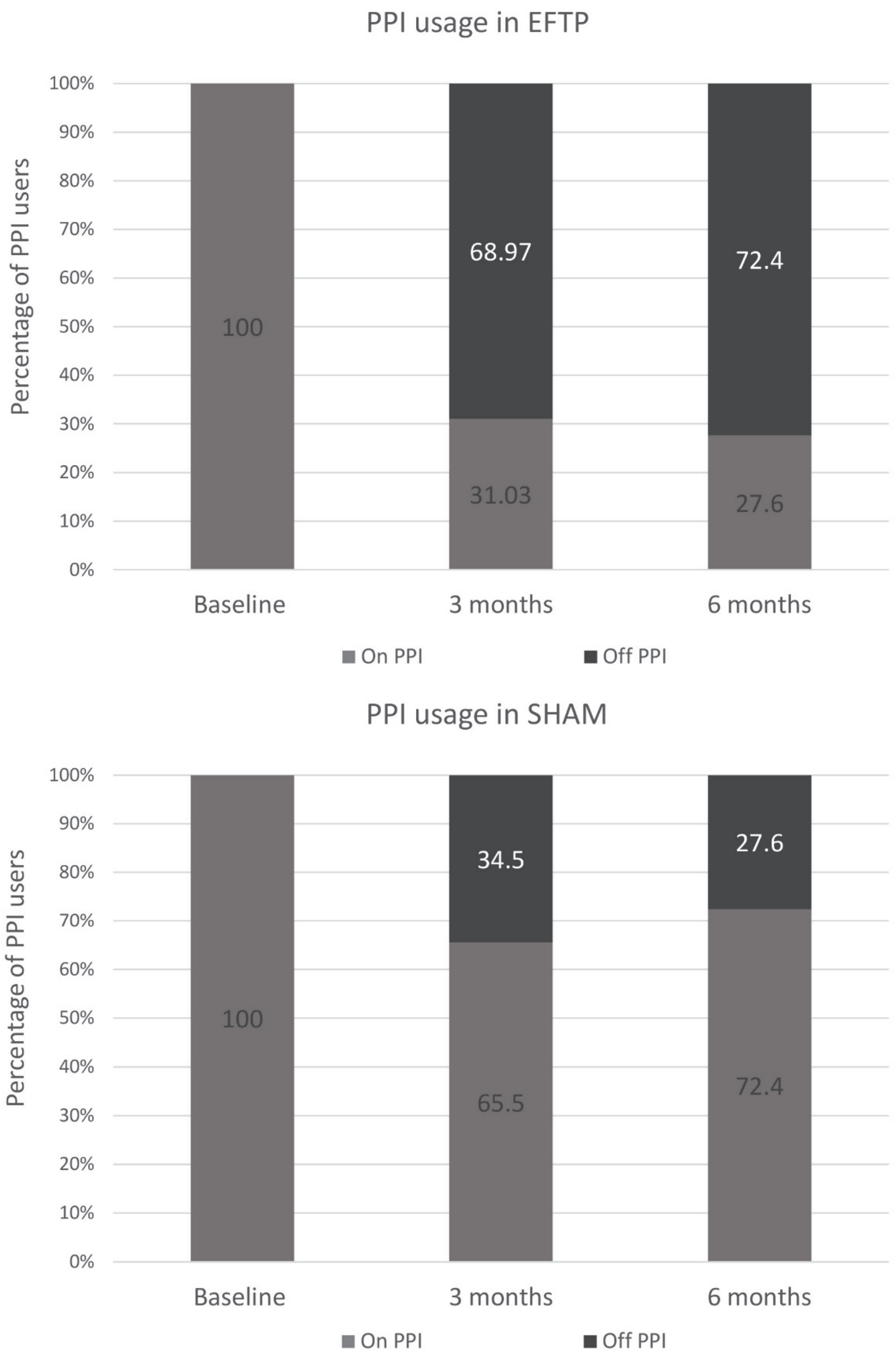


 | © 2023. Thieme. All rights reserved.

Supplementary material

**Appendix 1s**

**Patient's Telephonic reminder for scheduled follow up**

This is a gentle reminder with regard to the follow up appointment at the hospital. We mark your appointment scheduled on <date> at < time> as previously scheduled and consider that you will be attending. In case if you wish to change the date or time please let us know regarding the same. We look forward to seeing you at your appointment.

Follow up 1
Telephonic reminder 1 (Date and Time) _______________
Telephonic reminder 2 (Date and Time) _______________
Telephonic reminder 3 (Date and Time) _______________

First follow up missed Yes/ No If yes by how many days __________
Any fresh signs at follow up
PPI use

Follow up 2
Telephonic reminder 1 (Date and Time) _______________
Telephonic reminder 2 (Date and Time) _______________
Telephonic reminder 3 (Date and Time) _______________

Second follow up missed Yes/ No If yes by how many days __________
Any fresh signs at follow up
PPI use

Follow up 3
Telephonic reminder 1 (Date and Time) _______________
Telephonic reminder 2 (Date and Time) _______________
Telephonic reminder 3 (Date and Time) _______________

Third follow up missed Yes/ No If yes by how many days __________
Any fresh signs at follow up
PPI use

Any unscheduled visits (Date) _____________ Reason ______________

Any unscheduled visits (Date) _____________ Reason ______________

 | © 2023. Thieme. All rights reserved.
